# Awareness and Perceptions towards the Role of Systemic Inflammation and High-Sensitivity C-reactive Protein as a Biomarker in Atherosclerotic Cardiovascular Disease and Chronic Kidney Disease: The Multinational FLAME-ASCVD Survey amongst Cardiologists

**DOI:** 10.5334/gh.1382

**Published:** 2024-12-26

**Authors:** Nikolaus Marx, Issei Komuro, Preethy Prasad, Juying Qian, José Francisco kerr Saraiva, Amir Abbas mohseni Zonoozi, Abhijit Shete, Alberico L. Catapano

**Affiliations:** 1 Department of Internal Medicine I – Cardiology, University Hospital, RWTH Aachen University, Germany; 2 The University of Tokyo/International University of Health and Welfare, Tokyo, Japan; 3 Novo Nordisk, Copenhagen, Denmark; 4 Zhongshan Hospital, Shanghai, China; 5 Pontifical Catholic University of Campinas, Sao Paulo, Brazil; 6 currently at Eli Lilly, Dubai, UAE; 7 Novo Nordisk Healthcare AG, Zürich, Switzerland; 8 University of Milano, Milano, Italy; 9 Multimedica IRCCS, Italy

**Keywords:** Atherosclerosis, inflammation, Cardiovascular diseases, kidney diseases, Risk Factors, C-reactive protein

Mortality and morbidity remain high despite improvement in atherosclerotic cardiovascular disease (ASCVD) management (1). Systemic inflammation (SI) contributes to increased cardiovascular (CV) risk in people with ASCVD and chronic kidney disease (CKD) (1,2). Elevated levels of high-sensitivity C-reactive protein (hsCRP), a marker of SI, are predictive for future cardiovascular events (1,3). Current guidelines recognize the association between SI and ASCVD risk, but further guidance on using hsCRP in ASCVD and CKD risk stratification is needed (4,5).

Systemic inFLAMmation and rolE of hsCRP as a biomarker in AtheroSclerotic CardioVascular Disease (FLAME-ASCVD; URL: https://www.clinicaltrials.gov; Unique identifier: NCT05755373) is a cross-sectional, noninterventional, multinational online survey-based study conducted amongst cardiologists in 10 countries (Table 1) between March 24 and May 15, 2023. The WCG Institutional Review Board granted the study an exemption; study participants provided informed consent. The main objective was to assess awareness and perceptions of cardiologists towards the role of SI in patients with ASCVD and CKD. Additionally, the survey-based study assessed perception and potential use of hsCRP as a biomarker to identify SI in patients with ASCVD and CKD in routine clinical practice and to identify unmet clinical needs, potential barriers, and opportunities to improve ASCVD management. Interventional cardiologists (IC) and general cardiologists (GC) were included if they treated ≥15 patients with ASCVD and CKD (any stage) per month and practiced for at least three years. To minimize bias, the specific study topic was not disclosed in the invitation, and the screener design ensured respondents did not know the purpose of the study until they met the eligibility criteria.

**Table 1:** Summary of primary findings in FLAME-ASCVD (Systemic inFLAMmation and rolE of hsCRP as a biomarker in AtheroSclerotic CardioVascular Disease).


BASELINE CHARACTERISTICS OF PARTICIPATING CARDIOLOGISTS			

	**Total** **(N = 589)**	**Interventional cardiologists** **(n = 241)**	**General cardiologists** **(n = 348)**

Mean number of patients with ASCVD and CKD seen/treated in typical month ± (SD)*	39.3 (30.3)	34.3 (27.1)	42.7 (31.9)

	**Total** **(N = 490)**	**Interventional cardiologists** **(n = 206)**	**General cardiologists** **(n = 284)**

Age in years, mean (SD)*	47.2 (9.1)	46 (7.9)	48.1 (9.8)

	**Total** **(N = 214)**	**Interventional cardiologists** **(n = 77)**	**General cardiologists** **(n = 137)**

**Mean time in practice, years ± (SD)**	16.4 (7.0)	15.9 (6.6)	16.7 (7.3)

	**Total** **(N = 602)**	**Interventional cardiologists** **(n = 247)**	**General cardiologists** **(n = 355)**

**Region, n (%)** ^†^			

*Europe, 241 (40)*			

France*	60 (10)	13 (5)	47 (13)

Germany	60 (10)	24 (10)	36 (10)

Italy	61 (10)	30 (12)	31 (9)

United Kingdom	60 (10)	18 (7)	42 (12)

*East Asia, 120 (20)*			

China	60 (10)	30 (12)	30 (8)

Japan	60 (10)	30 (12)	30 (8)

*Asia Pacific, 121 (20)*			

Australia	60 (10)	30 (12)	30 (8)

India	61 (10)	30 (12)	31 (9)

*Latin America, 60 (10)*			

Brazil*	60 (10)	13 (5)	47 (13)

*Middle East, 60 (10)*			

Saudi Arabia	60 (10)	29 (12)	31 (9)

**Practice type, n (%)** ^†^			

Public (hospital, medical center, clinical practice)	297(49)	126 (51)	171 (48)

Private (hospital, clinical practice)	227 (38)	76 (31)	151 (43)

Voluntary, non-profit hospital University hospital*	78 (13)	45 (18)	33 (9)

**Practice setting, n (%)** ^†^			

Urban	540 (90)	218 (88)	322 (91)

Suburban	52 (9)	24 (10)	28 (8)

Rural	10 (2)	5 (2)	5 (1)

**Sex, n (%)** ^†^			

Male*	494 (82)	215 (87)	280 (79)

Female*	96 (16)	30 (12)	67 (19)

Prefer Not to Answer	<1 (2)	2 (1)	7 (2)

	**Total** **(N = 585)**	**Interventional cardiologists** **(n = 238)**	**General cardiologists** **(n = 347)**

**Mean number of patients seen/treated per typical month ± (SD)**			

Total number of patients, any condition*	321.4 (200.4)	297.3 (195.2)	337.9 (202.4)

	**Total** **(N = 535)**	**Interventional cardiologists** **(n = 224)**	**General cardiologists** **(n = 311)**

**Mean number of patients seen/treated per typical month with the following conditions ± (SD)**			

ASCVD^‡^	91.7 (79.9)	87.0 (89.8)	95 (72.0)

Heart failure*	51.7 (42.5)	41.3 (37.7)	59.3 (44.2)

Arrhythmia*	39.9 (29.9)	33.9 (29.6)	44.1 (29.4)

AMI	34.4 (31.2)	33.9 (28.2)	34.7 (33.2)

Cardiomyopathy*	31.4 (29.6)	28.3 (29.3)	33.5 (29.6)

Valvular disease*	32.8 (27.0)	28.5 (24.1)	35.8 (28.5)

Cerebrovascular disease*	28.1 (27.4)	24.0 (24.5)	31.0 (29.0)

PAD	26.4 (22.4)	25.2 (23.2)	27.3 (21.8)

Aortic disease	20.6 (19.6)	19.7 (20.9)	21.2 (18.6)

DVT/PE	17.7 (17.2)	17.8 (18.5)	17.6 (16.3)

Pericardial disease	13.9 (14.1)	13.5 (14.0)	14.2 (14.2)

	**Total** **(N = 571)**	**Interventional cardiologists** **(n = 232)**	**General cardiologists** **(n = 339)**

**Mean number of patients with ASCVD**^†^ **seen/treated in typical month by type ± (SD)**			

Coronary heart disease	65.5 (62.2)	60.6 (68.1)	69.0 (57.6)

Cerebrovascular disease*	21.7 (21.4)	17.9 (19.8)	24.4 (22)

**Risk factors of ASCVD discussed with patients** ^§^			

Risk Factor (%)	**Total** **(N = 601)**	**Interventional cardiologists** **(n = 247)**	**General cardiologists** **(n = 354)**

Hypertension*	88	85	91

Hyperlipidemia*	82	74	87

Lifestyle habits (diet, exercise)*	80	75	83

Hyperglycemia (both diabetes and pre-diabetes)	78	76	79

Overweight or obesity*	78	69	84

Impact of tobacco use	75	71	77

Risk factors for CAD and renal disease	55	51	58

CKD	56	58	55

Genetics/family history	54	52	55

Systemic inflammation	43	41	45

**TOP REASONS FOR CONSIDERING OR NOT CONSIDERING SYSTEMIC INFLAMMATION IN THE MANAGEMENT OF PATIENTS WITH ASCVD AND CKD** ^||^			

	**Total**	**Interventional cardiologists**	**General cardiologists**

**Reasons to consider systemic inflammation (%)**	**N = 602**	**n = 247**	**n = 355**

How aggressively to treat ASCVD	60	63	58

Lifestyle recommendations	49	44	52

How aggressively to treat CKD	44	44	44

**Reasons to not consider systemic inflammation (%)**	**N = 275**	**n = 108**	**n = 167**

Systemic inflammation would not change how I manage/treat	56	52	58

There are no available medications to treat systemic inflammation*	48	56	44

Systemic inflammation is a less useful indicator than other laboratory measures	24	21	25

**CARDIOLOGISTS’ ATTITUDES (% AGREE/STRONGLY AGREE) TOWARDS ROLE OF SYSTEMIC INFLAMMATION IN ASCVD AND CKD** ^#^			

Agree/Strongly agree (%)	**Total** **(N = 602)**	**Interventional cardiologists** **(n = 247)**	**General cardiologists** **(n = 355)**

Ongoing chronic inflammation is an important contributor to the risk of recurrent cardiovascular event	73	72	73

I believe systemic inflammation is a risk factor to develop ASCVD	71	70	72

Systemic inflammation is one the key drivers for cardiovascular events in patients with ASCVD and CKD	64	63	65

I would like to learn more about the role of systemic inflammation in ASCVD	62	61	63

Residual inflammatory risk still persists even with availability of evidence-based preventive cardiovascular therapies for ASCVD with CKD patients at risk	61	59	63

A lack of treatment options is the greatest unmet need facing patients with ASCVD and CKD	57	61	55

TOP THREE REASONS FOR CONSIDERING OR NOT CONSIDERING HSCRP TESTING TO IDENTIFY SI IN PATIENTS WITH ASCVD AND CKD**			

	**Total** **N = 602**	**Interventional cardiologists** **n = 247**	**General Cardiologists** **n = 355**

**Reasons to consider hsCRP, ranked 1 to 3 (%)**			

hsCRP will influence my clinical decisions*	43	48	39

Proven clinical efficacy	36	34	36

Is widely used for diagnosing inflammation*	34	28	38

**Reasons to not consider hsCRP (%)**			

There are not any available treatments; will not change clinical outcomes	26	27	26

hsCRP variability	23	25	21

hsCRP will not influence my practice	22	22	22


*Statistical significance was observed between the groups IC and GC, p < 0.05. ^†^Percentages may not sum to 100% due to rounding. ^‡^ASCVD defined as a patient who has had one or more of the following in the last 5 years: 1) Coronary heart disease defined as at least one of the following: documented history of MI, prior coronary revascularization procedure, or ≥50% stenosis in major epicardial coronary artery documented by cardiac catheterization or CT coronary angiography; 2) Cerebrovascular disease defined as at least one of the following: prior stroke of atherosclerotic origin, prior carotid artery revascularization procedure, or ≥50% stenosis in carotid artery documented by X-ray angiography, MR angiography, CT angiography or Doppler ultrasound; 3) Symptomatic peripheral artery disease defined as at least one of the following (or as locally defined): intermittent claudication with an ankle-brachial index (ABI) ≤ 0.90 at rest, intermittent claudication with a ≥50% stenosis in peripheral artery (excluding carotid) documented by X-ray angiography, MR angiography, CT angiography or Doppler ultrasound, prior peripheral artery (excluding carotid) revascularization procedure, or lower extremity amputation at or above ankle due to atherosclerotic disease (excluding e.g., trauma or osteomyelitis). ^§^Responses were to the survey question: When discussing the risk of ASCVD with your patients, which of the following factors do you most often discuss? ^||^Response to the survey questions: A. Which aspects of management/treatment of patients with both ASCVD and CKD are influenced by the results of the test you order to measure systemic inflammation? B. In cases which you do not consider systemic inflammation in decision-making for your patients with both ASCVD and CKD, what are the reason(s)? ^#^Responses were to the survey question: To what extent do you agree or disagree with the following statements (on a scale of 1 [strongly disagree] or 7 [strongly agree]; agree/strongly agree refer to a pooled score of 6 and 7). **Responses were to the survey questions: A. Which of the following, if any, are the top 3 reasons you would consider hsCRP testing to diagnose systemic inflammation in an ASCVD patient with CKD? B. Which of the following, if any, are reasons why you would not use hsCRP testing to diagnose systemic inflammation in an ASCVD patient with CKD? Abbreviations: AMI, acute myocardial infarction; ASCVD, atherosclerotic cardiovascular disease; CAD, coronary artery disease; CKD, chronic kidney disease; DVT/PE, deep vein thrombosis/pulmonary embolism; hsCRP, high-sensitivity C-reactive protein; PAD, peripheral artery disease; SD, standard deviation; SI, systemic inflammation.

General cardiologists were defined as heart failure specialists, clinical or general cardiologists, cardiac imaging specialists, preventive cardiologists, and cardiac rehabilitation specialists. Interventional cardiologists were identified through self-identification by participants. Administration of the survey was online; recruiting was conducted either online or by telephone. The invitation included general information about the survey and a link to a secure online platform to self-administer an initial set of screening questions to assess eligibility. Descriptive statistical analyses (means, frequencies) were performed using Q Research Software for Windows 23 (A Division of Displayr, Inc., New South Wales, Australia). Tests of differences (chi square, t-tests) within respondent types were performed using Q Research Software tables. Statistical significance was set at p < 0.05, using two-tailed tests. Data is presented as number and percentage for categorical variables, and continuous data is expressed as the mean ± standard deviation (SD) unless otherwise specified. The sample was targeted for demographic representativeness based on a sample size of N = 602 (distributed as 60–61 per country). Without using a finite population correction, the maximum margin of error for this study was 4% at a 95% confidence level. Pre-test interviews of approximately 45 minutes in duration were conducted among cardiologists (n = 12) across a subset (six) of the participating countries to pilot test and enhance the quality of the survey instrument; minor modifications were made for clarity and relevance.

Sample characteristics of the participating cardiologists and key survey data are presented in Table 1. A total of 602 cardiologists across five regions qualified for and completed the survey [IC, 247 (41%); GC, 355 (59%)]. Most participants reported commonly discussing risk factors for ASCVD with their patients, including hypertension (88%), hyperlipidemia (82%), lifestyle habits like diet/exercise (80%), hyperglycemia (78%), and obesity/overweight (78%). CKD was discussed by 56%, and SI was reported by 43% of cardiologists. Cardiologists acknowledged that limited awareness of the role of SI in ASCVD (42%) and lack of effective treatment options for SI (41%) are common unmet needs faced by patients with ASCVD and CKD.

Sixty-four percent of cardiologists (n = 386) acknowledged their intention to test for SI for management of patients with ASCVD, 41% of whom indicated the results could influence their decision to initiate anti-inflammatory treatment. Of those not assessing SI, 36% stated lack of medication for SI as one of the main reasons. Overall, 64% agreed/strongly agreed that SI is one of the drivers for CV events in patients with ASCVD and CKD, and 61% acknowledged that residual inflammatory risk persists in spite of evidence-based treatments for ASCVD-CKD. A majority (62%) agreed that they would like to learn more about the role of SI in ASCVD.

When a patient was diagnosed with CKD prior to/at the same time as their ASCVD, 47% (n = 571) of cardiologists tested hsCRP in patients with ASCVD. However, when asked via an unaided question (where respondents were not given pre-defined response choices) what comes to mind when thinking about a laboratory test typically used to identify SI, hsCRP testing was mentioned by 24%; C-reactive protein (i.e., standard CRP) was mentioned by 59%, procalcitonin by 29%, and erythrocyte sedimentation rate by 25% (p = not significant). Furthermore, only 8% considered hsCRP testing as a standalone measurement tool for SI without being accompanied by other inflammatory indicators. Factors reported as most influential for establishing hsCRP testing as the standard of care for patients with ASCVD and CKD were the need for guideline recommendations (45%), proven clinical efficacy (45%), and availability of treatments for SI (38%).

In this survey, SI was less commonly discussed with patients than traditional CV risk factors. However, respondents perceived SI as a CV risk factor and acknowledged it was a driver for CV events and contributes to the risk of recurrent CV events. A strength of this study is the collection of real-world data across multiple regions worldwide from both interventional and general cardiologists, adding to the limited literature. Key limitations include potential participant recall bias due to the self-reported nature of the study and generalizability due to the use of an online panel.

This study highlights patient unmet needs and cardiologists’ perceptions and awareness of SI. The study also underpins the need for education initiatives, guideline recommendations, and further research on these important clinical topics. There is a need for greater understanding of the role of SI and use of hsCRP as a biomarker to aid clinicians in making appropriate treatment choices for patients with ASCVD and CKD.

## Graphical Abstract

**Figure:**
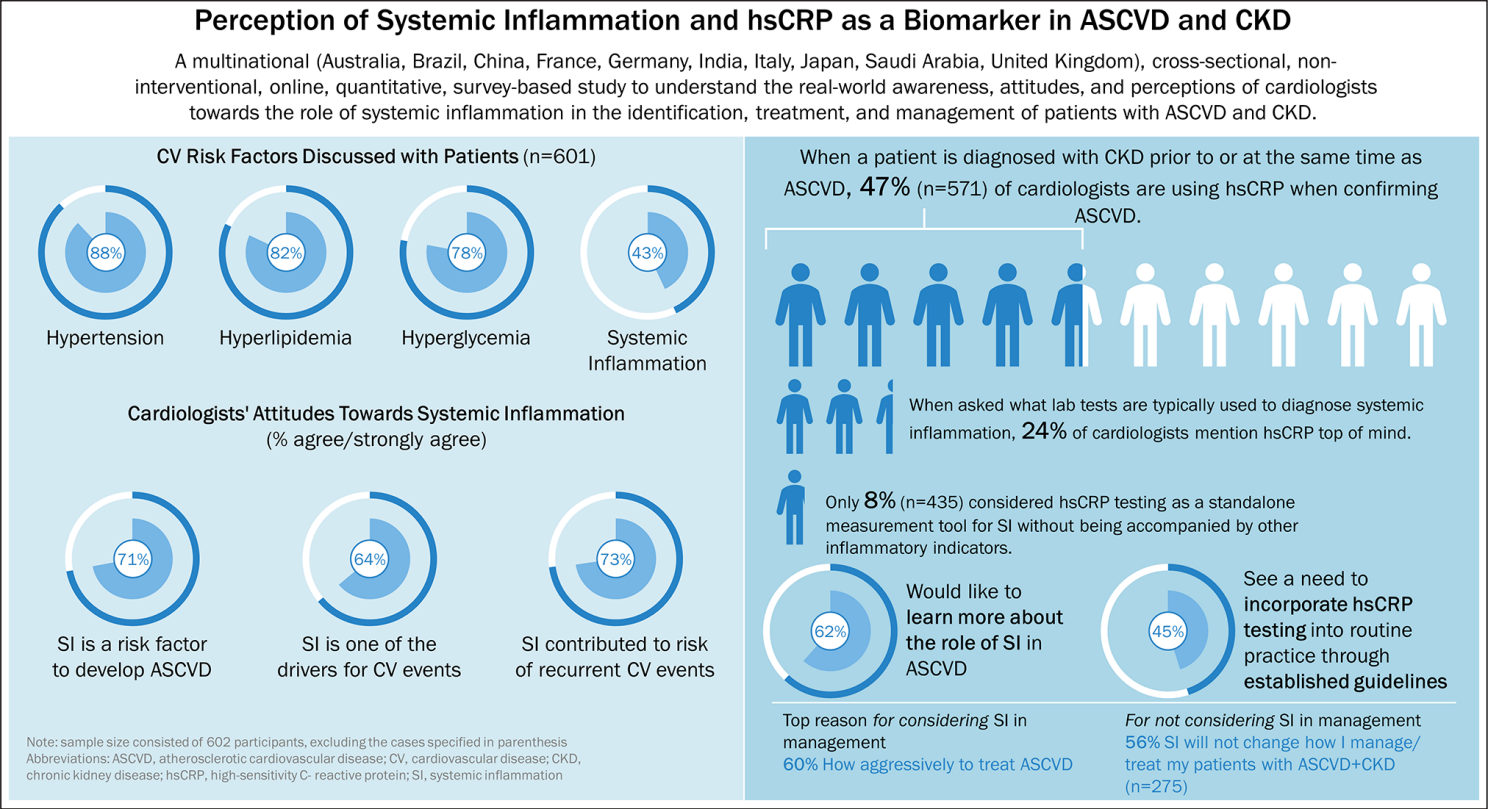


## Data Accessibility Statement

All data supporting the conclusions of these analyses are presented in the research letter. Details of additional data can be obtained from the corresponding author upon reasonable request.

## Additional File

The additional file for this article can be found as follows:

10.5334/gh.1382.s1 Supplementary Material. Survey.

## Prior Presentation

Some of the data from this study was presented as a poster at the American Heart Association Scientific Sessions November 11–13, 2023, in Philadelphia, Pennsylvania, USA.
