# Supplementary material for: Awareness and Perceptions towards the Role of Systemic Inflammation and High-Sensitivity C-reactive Protein as a Biomarker in Atherosclerotic Cardiovascular Disease and Chronic Kidney Disease: The Multinational FLAME-ASCVD Survey amongst Cardiologists
Source: Glob Heart. 2024 Dec 26;19(1):98. doi: 10.5334/gh.1382 (PMC11673585; doi:10.5334/gh.1382)
Supplement: Supplementary Material. — Survey. [file gh-19-1-1382-s1.pdf]

KJT Group, Inc.
777 Canal View Blvd
Suite 1500
Rochester, NY 14623

FLAME-ASCVD

A multinational survey-based study to understand the real-world awareness and perceptions of systemic inFLAMmation and rolE of hsCRP as a biomarker in patients with AtheroSclerotic CardioVascular Disease and chronic kidney disease among cardiologists

Survey

March 23, 2023

Lynn Clement
lynn@kjtgroup.com
585-582-5044

Rebecca Casbeer
rebeccac@kjtgroup.com
585.582.5103

Bridget Kelly
bridgetk@kjtgroup.com
585-582-5064

Isabella Barbagallo
isabellab@kjtgroup.com
585-582-5091

Primary Objective: To understand the real-world awareness, attitudes and perceptions of cardiologists towards the role of systemic inflammation in the identification, treatment and management of patients with ASCVD and CKD.

Secondary Objectives:

- To understand the real-world acceptance, perception and potential usage patterns for assessing high-sensitivity CRP (hsCRP) to identify systemic inflammation in patients with ASCVD and CKD in routine clinical practice.
- To identify unmet clinical needs for assessing systemic inflammation and/or potential barriers and opportunities to improve ASCVD management and treatment while leveraging hsCRP $\geq$2 mg/L.

SECTION S: SCREENING QUESTIONS

ALL RESPONDENTS
S0 Thank you for taking the time to participate in this important research. For your convenience we are offering this survey in multiple languages. Please select a language that you are most familiar with.

1. English (United States)
2. English (United Kingdom)
3. English (Australia)
4. Italian
5. Portuguese (Brazil)
6. Arabic (Saudi Arabia)
7. Japanese
8. German
9. French
10. Mandarin
11. Hindi

ALL RESPONDENTS
S1 Thank you for your interest in this survey. We appreciate your willingness to participate in this important research on healthcare topics.

Before participating, please review the following information carefully before your participation:

Participant and Agreement
to Take Part Form

Study title: Systemic Inflammation and hsCRP Research
Principal investigator: Dan Wasserman
Contact number: 1-585-582-5103
Ethics committee: WCG IRB

Research sponsor contact information: Novo Nordisk A/S, Novo Allé, 2880 Bagsvaerd, Denmark

1. **Study background and objective:**

- The survey is sponsored by Novo Nordisk IO-SO and led by CVD Scientific and Medical Affairs. The principal investigator will be Dan Wasserman. The planned survey duration is between 08 Feb to 8 May 2023.
- This study would assess cardiologists' perceptions towards systemic inflammation as an independent cardiovascular risk factor.
- This study will be reviewed by a Central IRB, **WCG Institutional Review Board (IRB),** located in the United States. The Ethics Committee is a dedicated organization for participant right protection.

2. **Study content, methods and procedures:**

- The purpose of this survey is to collect information for scientific research and to better understand the role of systemic inflammation in identification, treatment and management of patients with ASCVD and CKD.

- Your responses to this survey will **help the sponsor (Novo Nordisk) understand the healthcare experiences of physicians and patients.**

- The survey aims to collect data from approximately **600 HCPs.**

- This study is a survey-based study without collection of laboratory data. The study is not related to any specific treatment options or pharmaceutical product.

- With your agreement of participation, **the survey will be administered online** (you will receive a QR code/link to be scanned/clicked for access to the questionnaire). Your answer will be used exclusively for survey purpose and will be kept confidential. You also authorize the Pecking Union Medical College Hospital and the research team to review your answers for scientific purpose.

- We expect, on average, it will take respondents like yourself **20 minutes** to complete this survey.

3. **Possible risks (or discomforts, inconveniences) and benefits (personal or social benefits):**

- Potential risks: There are no costs to you for your participation. Given its questionnaire-based characteristics, the survey will not interfere with your personal life or have impact on your health status, and will not involve compensation, indemnity and medical measures for injuries that exceed the minimum risk. If you have any questions during the survey, you are encouraged to consult with the investigator.

- Potential benefits: If you qualify and complete this survey, you will receive compensation for time spent in line with approved local fair market value. Your response to this survey will help us identify the role of systemic inflammation in identification, treatment and management of patients with ASCVD and CKD. We would like to thank you for your participation and contribution to scientific progress!

4. **Survey contact:**

- You have the right to consult investigator about questions, complaints, concerns, for a research-related problems, or if you would like to follow-up on these points or need additional support through phone number 1-585-582-5103.

- You also have the right to consult the Ethics Committee about your rights or the study related risks through phone number 1-855-818-2289.

5. **Right to withdraw from study:**

- **Your participation in the study is completely voluntary,** and you may choose to not participate or stop participating at any time (withdraw your participation) without any penalty or loss of benefits to which you are otherwise entitled.

- You can choose to stop participating after completion of the questionnaire and withdraw your participation at any time by contacting 1-585-582-5103.

- Your unwillingness to participate or continue to participate in this study for any reason will not affect your rights in any way. You may choose to withdraw your participation at any time.

6. **Indemnity for study:** This study is survey-based and you will only fill in the questionnaires during the whole study period without damage to their health, so the treatment compensation and indemnity will not be provided.

7. **Legal Basis and Confidentiality:**

KJT Group Inc., the data processor, will collect personal information from you (such as your age, gender, ASCVD patient profile and diagnosis, testing practices, drivers and barrier to using testing, and ASCVD management attitudes) to set-up, conduct survey and analyze the results of the survey, on behalf of Novo Nordisk, the data controller.

We collect your information for several reasons. When doing so we follow the relevant privacy laws:

- We collect the information for scientific research and to better understand. The legal basis for this is our legitimate interests to understand and develop new medicines and scientific research.
- We may share knowledge and information gained from the survey with the scientific community. The legal basis for this is our legitimate interests and for reasons of public interest in the area of public health.
- We may share knowledge and information gained from the survey with health authorities. The legal basis for this is to meet the legal obligations of Novo Nordisk and for reasons of public interest in the area of public health. It will not be possible to identify you in any future presentations and publications resulting from this work.

- Any information you provided in this survey will be used only for this study and will be kept strictly confidential and never be connected with your name.

- Your responses will be grouped together with other responses and a summary of the results will be made publicly available in a peer-reviewed scientific journal publication once the study has finished. However, **your name or any other identifiable information will never be included in the report, publication, or identified to the sponsor**.

- All questionnaires collected online will be quality-controlled by professionals. All items in the questionnaire will be stored in a unique questionnaire database established for this study. A de-identified ID will be assigned to your questionnaire and investigators can only see the individual IDs.

- Your questionnaire will be stored electronically on a designated computer which is not used for other purposes and has a password only available to investigators.

- The study information collected about you will not be looked at by people who are not authorized to do so. To make sure the study is done correctly and to check the results, the following people will be able to see your study information.

    - KJT Group, Inc. staff responsible for conducting the survey have access to your identifiable information.

    - Novo Nordisk staff will receive a copy of the summary results where all personal identifiable information has been removed. Therefore, you will not be directly identified.

- Your responses will be transferred to and **stored on secure servers in the United States** for at least 5 years after the study results are summarized. Novo Nordisk will store the summary results (de-identified) from the survey for a minimum of 25 years after the study has ended as the information is considered to have scientific value and be relevant for safety reasons.

- **Your directly identifiable personal information will be destroyed within 12 months of data collection**; however, a permanent record of this agreement will be maintained.

- If you do not qualify for the study, your personal data will be stored electronically and erased no later than 12 months after the end of data collection.

- If your information is transferred out of the European Union/European Economic Area, such transfer of personal data will either be subject to Novo Nordisk binding corporate rules or the European Commission's standard contractual clauses. You can request a copy of the measures applying to the transfer of personal data by contacting privacy@novonordisk.com.

- The data protection law in the United States will be followed when processing personal information.

- Applicable data protection laws will be followed when processing personal information. (Including the EU General Data Protection Regulation [GDPR]).

This research was reviewed by WCG IRB. An IRB is a group of people who perform independent review of research studies. You may talk to them at IRB contact number1-855-818-2289.

A description of this survey-based study will be available on http://www.ClinicalTrials.gov. This Web site will not include information that can identify you. At most, the Web site will include a summary of the results. You can search this Web site at any time.

8. Information about your rights and ethics

You have the right to:

- see and get a copy of your information

- ask to limit the use of your information
- ask to have information corrected.

You can contact the Principal Investigator for more information about your rights.
If you have any questions, concerns, or complaints as to how Novo Nordisk is using your personal information, you can contact your Principal Investigator. You may also contact Novo Nordisk's Data Protection Officer at privacy@novonordisk.com. However, please note that only the Principal Investigator has access to your personal information. As Novo Nordisk cannot identify you, Novo Nordisk will only be able to provide you information about the general study set-up. If you would like to get information specific about you, please first contact the study Principal Investigator.

The French Data Protection Authority ('Commission nationale de l'informatique et des libertés (CNIL)) is responsible for making sure that laws about personal information are followed in France. For more information about your rights, or if you wish to make a complaint, you can contact +33 1 53 73 22 22.

The German Data Protection Authority (Die Bundesbeauftragte für den Datenschutz und die Informationsfreiheit) is responsible for making sure that laws about personal information are followed in Germany. For more information about your rights, or if you wish to make a complaint, you can contact +49 228 81995 0.

The Danish Data Protection Authority (Datatilsynet) is responsible for making sure that laws about personal information are followed in India For more information about your rights, or if you wish to make a complaint, you can contact +45 33 1932 00.

The Italian Data Protection Authority (Garante per la protezione dei dati personali) is responsible for making sure that laws about personal information are followed in Italy. For more information about your rights, or if you wish to make a complaint, you can contact +39 06 69677 1.

The Information Commissioner's Office is responsible for making sure that laws about personal information are followed in the United Kingdom. For more information about your rights, or if you wish to make a complaint, you can contact +44 1625 545 745.

Acknowledgement of Participant:

I have read and fully understand the contents above, and have carefully considered my rights, risks and benefits of participation. I voluntarily participate in this study, and I am willing to cooperate with the investigators. I also declare that I can withdraw from this study at any time and for any reason without loss of any legal rights.

Do you agree to these terms and wish to continue with the survey?

1. Yes
2. No

CONSENTS TO PARTICIPATE (S1r1)
S5 Which of the following best describes your current role?

1. Physician
2. Nurse
3. Pharmacist (Pharm.D.)
4. Other

PHYSICIAN (S5r1)
S10 Which of the following is your primary medical specialty?

1. Cardiology
2. Family Practice / General Practice
3. Internal Medicine
4. Other

SPECIALTY IN INTERNAL MEDICINE (S10r3)
S12 Which of the following is your primary focus in Internal Medicine?

1. Cardiology **CONTINUE**
2. Critical Care
3. Endocrinology
4. Other, please specify:

CARDIOLOGY SPECIALTY (S10r1 OR S12r1)
S15 Which of the following best describes the primary type of cardiology of your practice?

1. Interventional cardiologist
2. Cardiac surgeon **END**
3. Heart failure specialist
4. Clinical / general cardiologist
5. Cardiac electrophysiologist **END**
6. Cardiac imaging specialist
7. Congenital heart specialist **END**
8. Cardio-oncologist **END**
9. Preventive cardiologist
10. Cardiac rehabilitation specialist
11. Other **END**

PHYSICIAN (S5r1)
S20 How many years have you been in practice beyond your residency or fellowship?

If you are still in your residency, are currently a fellow, or have not been in practice for at least one year, please enter "0" (zero).

1. |_|_|_| # of years

PHYSICIAN (S5r1)
S25

UK (P1r1)
S25A In what country is the practice where you spend most of your time located?

1. England
2. Northern Ireland
3. Scotland
4. Wales
99. I do not practice in the UK

ITALY (P1r2)
S25B In what region is the practice where you spend most of your time located?

1. Abruzzo
2. Aosta Valley
3. Apulia
4. Basilicata
5. Calabria
6. Campania
7. Emilia-Romagna
8. Friuli-Venezia Giulia
9. Lazio
10. Liguria
11. Lombardy
12. Marches
13. Molise
14. Piedmonte
15. Sardinia
16. Sicily
17. Trentino-South Tyrol
18. Tuscany
19. Umbria
20. Veneto
99. I do not practice in Italy

GERMANY (P1r3)
S25C In which state is the practice where you spend most of your time located?

1. Baden - Wurttemberg Map
2. Bavaria (Bayern) Map
3. Berlin

4. Brandenburg
5. Bremen
6. Hamburg
7. Hesse (Hessen)
8. Lower Saxony (Niedersachsen)
9. Mecklenburg-Vorpommern
10. North Rhine - Westphalia (Nordrhein-Westfalen)
11. Rhineland - Palatinate (Rheinland-Pfalz)
12. Saarland
13. Saxony (Sachsen)
14. Saxony - Anhalt (Sachsen-Anhalt)
15. Schleswig-Holstein
16. Thuringia (Thueringen)
99. I do not practice in Germany

BRAZIL (P1r4)

S25D In what province or territory is the practice where you spend most of your time located?

1. Acre
2. Alagoas
3. Amapá
4. Amazonas
5. Bahia
6. Ceará
7. Distrito Federal
8. Espírito Santo
9. Goiás
10. Maranhão
11. Mato Grosso
12. Mato Grosso do Sul
13. Minas Gerais
14. Pará
15. Paraíba
16. Paraná
17. Pernambuco
18. Piaui
19. Rio de Janeiro
20. Rio Grande do Norte
21. Rio Grande do Sul
22. Rondônia
23. Roraima
24. Santa Catarina
25. São Paulo
26. Sergipe
27. Tocantins
99. I do not practice in Brazil

KSA (P1r5)
S25E In what district is the practice where you spend most of your time located?

1. Qassim
2. Riyadh
3. Tabuk
4. Madinah
5. Makkah
6. Northern Borders
7. Jawf
8. Ha'il
9. Bahah
10. Jizan
11. ‘Asir
12. Najran
13. Eastern Province
99. I do not practice in Saudi Arabia

JAPAN (P1r6)
S25F In what region/prefecture is the practice where you spend most of your time located?

1. Aichi
2. Akita
3. Aomori
4. Chiba
5. Ehime
6. Fukui
7. Fukuoka
8. Fukushima
9. Gifu
10. Gunma
11. Hiroshima
12. Hokkaidō
13. Hyōgo
14. Ibaraki
15. Ishikawa
16. Iwate
17. Kagawa
18. Kagoshima
19. Kanagawa
20. Kōchi
21. Kumamoto
22. Kyōto
23. Mie
24. Miyagi
25. Miyazaki
26. Nagano
27. Nagasaki

28. Nara
29. Niigata
30. Ōita
31. Okayama
32. Okinawa
33. Ōsaka
34. Saga
35. Saitama
36. Shiga
37. Shimane
38. Shizuoka
39. Tochigi
40. Tokushima
41. Tōkyō
42. Tottori
43. Toyama
44. Wakayama
45. Yamagata
46. Yamaguchi
47. Yamanashi
99. I do not practice in Japan

AUSTRALIA (P1r7)

S25G In which state or territory is the practice where you spend most of your time located?

1. Australian Capital Territory
2. New South Wales
3. Northern Territory
4. Queensland
5. South Australia
6. Tasmania
7. Victoria
8. Western Australia
99. I do not practice in Australia

CHINA (P1r8)

S25H In what province is the practice where you spend most of your time located?

1. Anhui
2. Beijing
3. Chongqing
4. Fujian
5. Gansu
6. Guangdong
7. Guangxi
8. Guizhou
9. Hainan
10. Hebei

11. Heilongjiang
12. Henan
13. Hubei
14. Hunan
15. Jiangsu
16. Jiangxi
17. Jilin
18. Liaoning
19. Inner Mongolia
20. Ningxia
21. Qinghai
22. Shaanxi
23. Shandong
24. Shanghai
25. Shanxi
26. Sichuan
27. Tianjin
28. Xinjiang
29. Xizang
30. Yunnan
31. Zhejiang
99. I do not practice in China

INDIA (P1r9)

S25I In which state or territory is the practice where you spend most of your time located?

1. Andhra Pradesh
2. Arunachal Pradesh
3. Assam
4. Bihar
5. Chhattisgarh
6. Goa
7. Gujarat
8. Haryana
9. Himachal Pradesh
10. Jharkhand
11. Karnataka
12. Kerala
13. Madhya Pradesh
14. Maharashtra
15. Manipur
16. Meghalaya
17. Mizoram
18. Nagaland
19. Odisha

20. Punjab
21. Rajasthan
22. Sikkim
23. Tamil Nadu
24. Telangana
25. Tripura
26. Uttar Pradesh
27. Uttarakhand
28. West Bengal
29. Andaman and Nicobar Islands
30. Chandigarh
31. Dadra & Nagar Haveli and Daman & Diu
32. Delhi
33. Jammu and Kashmir
34. Lakshadweep
35. Puducherry
36. Ladakh
99. I do not live in India

FRANCE (P1r10)
S25J In what region is the practice where you spend most of your time located?

1. Auvergne - Rhône-Alpes
2. Bretagne (Brittany)
3. Bourgogne - Franche-Comté
4. Corse (Corsica)
5. Centre - Val de Loire
6. Grand Est (Alsace, Champagne, Lorraine)
7. Hauts de France (Nord Pas-de-Calais - Picardie)
8. Ile de France (Paris)
9. Nouvelle Aquitaine (Aquitaine, Poitou-Charentes, Limousin)
10. Normandie
11. Occitanie (Midi-Pyrénées, Languedoc)
12. Pays de la Loire
13. Provence - Cote d'Azur

PHYSICIAN (S5r1)
S30 Which of the following best describes your primary practice setting? If you practice in more than one location, please select the option that represents where you spend the majority of your time.

1. Public hospital
2. Voluntary, non-profit hospital University hospital
3. Private hospital

4. Private practice
5. Public medical center/clinic
6. Other

PHYSICIAN (S5r1)
S40 In a typical month, approximately how many total adult patients (age 18 or older) did you personally see/treat across all conditions and all care settings (hospitals, outpatient clinics, etc.)?

Your best estimate is fine.

1. Patient(s) in a typical month |_|_|_|_|

PHYSICIAN (S5r1)
QC1 For quality control purposes, please select "slightly happy" from the list of options below.

1. Very unhappy
2. Slightly unhappy
3. Neutral
4. Slightly happy
5. Very happy

TREATS AT LEAST 1 PATIENT (S40>0)
S45 Considering the [TOTAL SEEN] adult patients you see/treat in a typical month, approximately how many of these patients are diagnosed with each of the following conditions?

Please use your best estimate. Your responses may sum to more than [INSERT S40] to account for patients with multiple conditions. Your responses may sum to less than [INSERT S40] to account for not all patients having the conditions listed.

Please consider a person with ASCVD as someone who has had one or more of the following in the last 5 years:

a) Coronary heart disease defined as at least one of the following:
i. Documented history of MI
ii. Prior coronary revascularization procedure
iii. ≥50% stenosis in major epicardial coronary artery documented by cardiac catheterization or CT coronary angiography

b) Cerebrovascular disease defined as at least one of the following:
i. Prior stroke of atherosclerotic origin
ii. Prior carotid artery revascularization procedure
iii. ≥50% stenosis in carotid artery documented by X-ray angiography, MR angiography, CT angiography or Doppler ultrasound

c) Symptomatic peripheral artery disease (PAD) defined as at least one of the following (or as locally defined):

i. Intermittent claudication with an ankle-brachial index (ABI) ≤ 0.90 at rest
ii. Intermittent claudication with a ≥50% stenosis in peripheral artery (excluding carotid) documented by X-ray angiography, MR angiography, CT angiography or Doppler ultrasound
iii. Prior peripheral artery (excluding carotid) revascularization procedure
iv. Lower extremity amputation at or above ankle due to atherosclerotic disease (excluding e.g., trauma or osteomyelitis).

1. Atherosclerotic cardiovascular disease (ASCVD)	|_|_|_|
2. Congenital heart disease	|_|_|_|
3. Cardiomyopathy	|_|_|_|
4. Valvular disease	|_|_|_|
5. Arrhythmia	|_|_|_|
6. Peripheral arterial disease (PAD)	|_|_|_|
7. Aortic disease	|_|_|_|
8. Pericardial disease	|_|_|_|
9. Cerebrovascular disease	|_|_|_|
10. Deep vein thrombosis (DVT) or Pulmonary embolism (PE)	|_|_|_|
11. Acute Myocardial Infarction	|_|_|_|
12. Heart failure	|_|_|_|

TREATS AT LEAST 1 ASCVD PATIENT (S45r1>0)

S47 In the typical month, out of the [TOTAL ASCVD PATIENTS] adult patients you saw/treated with **ASCVD**, approximately how many fall into each of the following categories?

Your response may add to greater than [TOTAL ASCVD PATIENTS] *to account for patients that suffer from more than one of the following. Your best estimate is fine*.

Coronary heart disease defined as at least one of the following:
i. Documented history of MI
ii. Prior coronary revascularization procedure
iii. ≥50% stenosis in major epicardial coronary artery documented by cardiac catheterization or CT coronary angiography
b) Cerebrovascular disease defined as at least one of the following:
i. Prior stroke of atherosclerotic origin
ii. Prior carotid artery revascularization procedure
iii. ≥50% stenosis in carotid artery documented by X-ray angiography, MR angiography, CT angiography or Doppler ultrasound
c) Symptomatic peripheral artery disease (PAD) defined as at least one of the following (or as locally defined):
i. Intermittent claudication with an ankle-brachial index (ABI) ≤ 0.90 at rest
ii. Intermittent claudication with a ≥50% stenosis in peripheral artery (excluding carotid) documented by X-ray angiography, MR angiography, CT angiography or Doppler ultrasound
iii. Prior peripheral artery (excluding carotid) revascularization procedure
iv. Lower extremity amputation at or above ankle due to atherosclerotic disease

(excluding e.g., trauma or osteomyelitis).

1. Persons with **Coronary heart disease** seen/treated in the typical month |_|_|_|_|
2. Persons with **Cerebrovascular disease** seen/treated in the typical month |_|_|_|_|
3. Persons with **Symptomatic peripheral artery disease (PAD) disease** seen/treated in the typical month |_|_|_|_|

TREATS AT LEAST 1 ASCVD PATIENT (S45r1>0)
S50 In the typical month, out of the [TOTAL ASCVD PATIENTS] adult patients you saw/treated with **ASCVD**, approximately how many **also have chronic kidney disease (CKD)**? Please consider a person with CKD as someone with an eGFR of 60+ with increased albuminuria levels (Stage 1 CKD) or an eGFR of <60 (Stage 2+ CKD).

Please consider all persons that have ASCVD and CKD, whether or not their ASCVD or CKD were discussed during their visit in the typical month.

Your best estimate is fine.

1. Persons with **ASCVD and CKD** seen/treated in the typical month |_|_|_|_|

SECTION 200: PATIENT PROFILING AND DIAGNOSIS

ALL QUALIFIED RESPONDENTS (S100r1)
Q200 You can now complete the full survey. Thank you for your responses thus far. The remainder of this survey should take approximately **17 minutes** to complete.

As a reminder, your responses to this survey are critical to the success of this research in helping the sponsor design new products/services to help you support your patients' needs. Your responses will be kept strictly confidential and only reported in combination with other respondents' data. In addition, you may be asked certain questions for quality control purposes.

ALL QUALIFIED RESPONDENTS (S100r1)
Q201 To begin, considering your adult patients who may be at risk of **ASCVD**, how often do you typically discuss their risk of **ASCVD**?

Please answer for your typical patient at risk, to the best of your ability.

As a reminder, for the purposes of this survey please consider a person with ASCVD as someone who has had one or more of the following in the last 5 years:
a) Coronary heart disease defined as at least one of the following:
i. Documented history of MI

ii. Prior coronary revascularization procedure
iii. ≥50% stenosis in major epicardial coronary artery documented by cardiac catheterization or CT coronary angiography

b) Cerebrovascular disease defined as at least one of the following:
i. Prior stroke of atherosclerotic origin
ii. Prior carotid artery revascularization procedure
iii. ≥50% stenosis in carotid artery documented by X-ray angiography, MR angiography, CT angiography or Doppler ultrasound

c) Symptomatic peripheral artery disease (PAD) defined as at least one of the following (or as locally defined):
i. Intermittent claudication with an ankle-brachial index (ABI) ≤ 0.90 at rest
ii. Intermittent claudication with a ≥50% stenosis in peripheral artery (excluding carotid) documented by X-ray angiography, MR angiography, CT angiography or Doppler ultrasound
iii. Prior peripheral artery (excluding carotid) revascularization procedure
iv. Lower extremity amputation at or above ankle due to atherosclerotic disease (excluding e.g., trauma or osteomyelitis).

1. Never
2. Sometimes (every few visits)
3. Often (almost every visit)
4. At every visit

DISCUSSES RISK OF ASCVD (Q201r2-4)

Q202 When discussing the risk of **ASCVD** with your patients, which of the following factors do you most often discuss?

Please select all that apply.

1. Aging
2. Overweight or obesity
3. Genetics / family history
4. CKD
5. Hyperglycaemia (Both diabetes mellitus and prediabetes mellitus)
6. Hypertension
7. Hyperlipidaemia
8. Impacts of alcohol use [ALWAYS SHOW BEFORE CODE 9]
9. NASH
10. Side effects of a medication for another condition
11. Lifestyle habits (i.e., diet, exercise)
12. Impacts of Tobacco use
13. Systemic inflammation
14. Cardiac enzymes
15. How to prevent risk factors that ASCVD can lead to such as carotid artery disease and renal artery disease
16. Sleep apnea
17. Hyperuricemia

18. Rheumatoid arthritis
96. Other, please specify:

ALL QUALIFIED RESPONDENTS (S100r1)
Q206 Considering your adult patients who may be diagnosed with **ASCVD**, how often do you typically discuss potential complications with their ASCVD?

1. Never
2. Sometimes (every few visits)
3. Often (almost every visit)
4. At every visit

ALL QUALIFIED RESPONDENTS (S100r1)
Q209 Thinking of your patients who have **ASCVD**, approximately what percentage are **diagnosed** in each of the following ways?

Your best estimate will do. Your answers must sum to 100%.

1. Percentage of patients diagnosed with ASCVD

1. I personally diagnose with ASCVD |_|_|_|%
2. I refer to someone else for diagnosis |_|_|_|%
3. Diagnosed by someone else without my involvement |_|_|_|%

ALL QUALIFIED RESPONDENTS (S100r1)
Q320 NEW PLACEMENT Thinking about the lab tests typically used in diagnosing **systemic inflammation**, what specific tests come to mind?

Please list up to 5 tests that come to mind, and please type only one name per box. If you are not aware of any specific names, please type "None" into the first box.

DIAGNOSES SOME PATIENTS WITH ASCVD (Q209r1>0)
Q210 When a patient comes to you with symptoms that may indicate **ASCVD**, what do you typically do to **confirm a diagnosis of ASCVD**?

Please select all that apply.

1. Review patient's clinical history
2. Patient's presentation of symptoms
3. Imaging (i.e., MRI, CT Scan, angiogram, ultrasound, non-invasive stress imaging)
4. Blood testing specific to indicators of ASCVD – lipids
5. Blood testing specific to indicators of ASCVD – HBA1c
6. Blood testing for inflammation – hsCRP
7. Blood testing for inflammation – CRP
8. Blood testing for inflammation – plasma viscosity (PV)

9. Blood testing <u>for inflammation</u> – erythrocyte sedimentation rate (ESR)
10. Blood testing for Troponin and other heart-related markers
11. Cardiac catheterization
12. Echocardiogram (Echo)
13. Electrocardiogram (ECG)
14. Nuclear stress test
15. Cardiovascular Risk Scores (e.g. Framingham, QRISK2, QRISK3, etc.)
16. Coronary calcium score
17. Refer to a specialist/another physician
96. Other, please specify:

ALL QUALIFIED RESPONDENTS (S100r1)
Q212 Now, for the remainder of today's survey, we want you to focus on your **patients who are currently diagnosed with <u>both</u> atherosclerotic cardiovascular disease (ASCVD) <u>and</u> Chronic Kidney Disease (CKD).**

For reference, earlier you mentioned you see/treat [TOTAL ASCVD?CKD PATIENTS] patients diagnosed with ASCVD <u>and</u> CKD in a typical month.

Please click continue to proceed.

ALL QUALIFIED RESPONDENTS (S100r1)
Q220 Approximately what percentage of your patients with **ASCVD <u>and</u> CKD** were:

Your responses must sum to 100%. Your best estimate will do.

1. Percentage of patients diagnosed with ASCVD <u>and</u> CKD

1. Diagnosed with ASCVD <u>prior</u> to being diagnosed with CKD	|_|_|_|%
2. Diagnosed with CKD <u>prior</u> to being diagnosed with ASCVD	|_|_|_|%
3. Diagnosed with ASCVD and CKD at the same time	|_|_|_|%
4. Not sure about sequence	|_|_|_|%

ALL QUALIFIED RESPONDENTS (S100r1)
Q221 Which group of your patients has more **systemic inflammation**?

For the purposes of this survey, please consider **systemic inflammation** to be an **hsCRP lab value of 2.0 – 10.0 mg/L.**

Please select one.

1. CKD patients
2. ASCVD patients
3. Patients with both ASCVD and CKD

SECTION 300: SUSPECTED ASCVD AND TESTING PRACTICES

HAVE PATIENTS WHOSE ASCVD PATIENTS DIAGNOSED AFTER OR AT SAME TIME AS CKD (Q220r2+r3>0)
Q301 Now, thinking specifically of your patients who were diagnosed with CKD **prior to or at the same time as their ASCVD**, what tests were used to confirm the **diagnosis of ASCVD**?

Please select all that apply.

1. Review patient's clinical history
2. Patient's presentation of symptoms
3. Imaging (i.e., MRI, CT Scan, angiogram, ultrasound, non-invasive stress imaging)
4. Blood testing specific to indicators of ASCVD – lipids
5. Blood testing specific to indicators of ASCVD – HBA1c
6. Blood testing for inflammation – hsCRP
7. Blood testing for inflammation – CRP
8. Blood testing for inflammation – plasma viscosity (PV)
9. Blood testing for inflammation – erythrocyte sedimentation rate (ESR)
10. Blood testing for Troponin and other heart-related markers
11. Cardiac catheterization
12. Echocardiogram (Echo)
13. Electrocardiogram (ECG)
14. Nuclear stress test
15. Cardiovascular Risk Scores (e.g. Framingham, QRISK2, QRISK3, etc.)
16. Coronary calcium score
17. Urinalysis (e.g., urine sediment, albuminuria)
18. Other, please specify:
19. None of these

USE hsCRP BLOOD TESTING FOR INFLAMMATION (Q301r6)
Q301A Thinking specifically of your patients who you used **hsCRP blood testing for systemic inflammation** to confirm the **diagnosis of ASCVD**, approximately how many **hsCRP blood tests** do you order per month?

Please enter a number.

___ hsCRP blood tests per **month**

USE hsCRP BLOOD TESTING FOR INFLAMMATION (Q301r6)
Q302 Thinking specifically of your patients who you used **hsCRP blood testing for systemic inflammation** to confirm the **diagnosis of ASCVD**, what other tests (if any) were **used in combination** with hsCRP?

Please select all that apply.

1. Imaging (i.e., MRI, CT Scan, angiogram, ultrasound, non-invasive stress imaging)
2. Blood testing specific to indicators of ASCVD – lipids
3. Blood testing specific to indicators of ASCVD – HBA1c
4. Blood testing for Troponin and other heart-related markers
5. Cardiac catheterization
6. Echocardiogram (Echo)
7. Electrocardiogram (ECG)
8. Nuclear stress test
9. Cardiovascular Risk Scores (e.g. Framingham, QRISK2, QRISK3, etc.)
10. Coronary calcium score
11. Urinalysis (e.g., urine sediment, albuminuria)
12. Other, please specify:
13. No other test was used in combination with blood testing for systemic inflammation

ALL QUALIFIED RESPONDENTS (S100r1)
Q307
What is the **minimum threshold of systemic inflammation** (diagnosed through hsCRP testing) that **impacts your decision-making** in **ASCVD** patient management?

1. hsCRP <2 mg/L
2. hsCRP 2 to <3 mg/L
3. hsCRP 3 to <5 mg/L
4. hsCRP 5 to <10 mg/L
5. hsCRP 10+ mg/L

ALL QUALIFIED RESPONDENTS (S100r14)
Q305 Which of the following statements best describe the **role systemic inflammation has** in how you **identify/diagnosis** and **treat/manage** patients that have both ASCVD and CKD?

For the purposes of this survey, please consider **systemic inflammation** to be an **hsCRP lab value of 2.0 – 10.0 mg/L.**

1. The **identification/diagnosis** of patients suspected of having ASCVD and known/suspected CKD
2. The **treatment and management** of patients with ASCVD and CKD

1. I **test** for systemic inflammation in my patients and use this **in my decision making**
2. I **don't consider** systemic inflammation markers **in decision making**
3. I **don't measure or test** for systemic inflammation markers directly

ALL QUALIFIED RESPONDENTS (S100r1)
QC2 For quality control purposes, please select "5" from the list of options below.

1. 1

2. 2
3. 3
4. 4
5. 5

DOES NOT CONSIDER INFLAMMATION (Q305c1 not r1 and Q305c2 not r1)
Q306 In cases which you **do not consider systemic inflammation** in decision-making for your patients with both **ASCVD and CKD,** what are the reason(s)?

For the purposes of this survey, please consider **systemic inflammation** to be an **hsCRP lab value of 2.0 – 10.0 mg/L.**

Please select all that apply.

1. It is **not easy to access the tests** needed to measure systemic inflammation
2. Systemic inflammation **would not change how I manage/treat** my patients with ASCVD and CKD
3. Systemic inflammation is a **less useful indicator** than other laboratory measures
4. Systemic inflammation is an **important risk** but I do not consider it because currently there are **no available medications** to treat it
5. Other, please specify:

ALL QUALIFIED RESPONDENTS (S100r1)
Q310 What percentage of your patients diagnosed with both **ASCVD and CKD** have also been diagnosed with **systemic inflammation** (hsCRP ≥2 mg/L)?

Your best estimate, based on the patients you test, will do.

1. [_|_|_]%
2. Not sure

ALL QUALIFIED RESPONDENTS (S100r1)
Q325 Which of the following **lab tests** for identifying **systemic inflammation** do you typically use? Please be sure to select those which you may have already indicated previously.

Please select all that apply.

1. C-reactive protein (standard CRP)
2. High-sensitivity CRP (hsCRP)
3. Erythrocyte Sedimentation Rate (ESR)
4. Procalcitonin (PCT)
5. Plasma viscosity (PV)
6. Interleukin 6 (IL-6)

7. Fibrinogen
8. Serum amyloid A
9. Haptoglobin
10. Secretory phospholipase A2
11. Lipoprotein(a)

12. Other, please specify:
13. I don't measure/assess inflammation through lab tests
14. Not aware of any lab tests

USE HIGH-SENSITIVITY CRP (hsCRP) (Q325r2)
Q330 When would you typically use hsCRP?

Please select all that apply.

1. To diagnose CKD
2. To diagnose ASCVD
3. Patients newly diagnosed with CKD
4. Patients newly diagnosed with ASCVD
5. Has a history of inflammatory conditions or diseases
6. To diagnose infections
7. Other, please specify:

USE HIGH-SENSITIVITY CRP (hsCRP) (Q325r2)
Q335 Which of the following, if any, do you use hsCRP in combination with when testing or measuring for **systemic inflammation**?

Please select all that apply.

1. Order a test of other inflammatory markers
2. Review patient's chart for other inflammatory markers
3. Review patient's history for an inflammatory condition/diseases (i.e., diabetes, obesity)
4. Other, please specify:
5. I do not use other tests or measurements

ALL QUALIFIED RESPONDENTS (S100r1)
Q336 How often do you measure/assess **systemic inflammation** through a lab test to guide management/treatment of patients with **ASCVD**?

For the purposes of this survey, please consider **systemic inflammation** to be an **hsCRP lab value of 2.0 – 10.0 mg/L.**

1. Weekly
2. Monthly
3. Bi-monthly (every other month)
4. Quarterly (every 3 months)
5. Bi-annually (every 6 months)
6. Yearly
7. Every 2 years
8. Every 3 years or less
9. Never

ALL QUALIFIED RESPONDENTS (S100r1)
Q340 How often do you measure/assess **systemic inflammation** through a lab test to guide management/treatment of patients with both **ASCVD** **and** **CKD**?

For the purposes of this survey, please consider **systemic inflammation** to be an **hsCRP lab value of 2.0 – 10.0 mg/L.**

1. Weekly
2. Monthly
3. Bi-monthly (every other month)
4. Quarterly (every 3 months)
5. Bi-annually (every 6 months)
6. Yearly
7. Every 2 years
8. Every 3 years or less
9. Never

ALL QUALIFIED RESPONDENTS (S100r1)
Q345 Which aspects of management/treatment of patients with both **ASCVD** **and** **CKD** are influenced by the results of the test you order to measure **systemic inflammation**?

For the purposes of this survey, please consider **systemic inflammation** to be an **hsCRP lab value of 2.0 – 10.0 mg/L.**

Please select all that apply.

1. How aggressively to treat CKD
2. How aggressively to treat ASCVD
3. Specific recommendations made about lifestyle modifications or weight loss
4. Which types of medications are prescribed (e.g. treatment with ARNi, ARB or ACEi in patients with HF
5. Which dosage of medication is prescribed
6. Referrals to specialists
7. Frequency of monitoring
8. Initiation of anti-inflammatory treatments such as colchicine

96. Other, please specify:

97. I would not change how I manage/treat my patients based on the systemic inflammation test results

SECTION 400: DRIVERS & BARRIERS TO USING HSCRP TESTING

ALL QUALIFIED RESPONDENTS (S100r1)
QC3 For quality control purposes, please select no.

1. Yes
2. No

ALL QUALIFIED RESPONDENTS (S100r1)
Q405a Which of the following, if any, are the **top 3** reasons you would consider **hsCRP testing** to diagnose **systemic inflammation** in an **ASCVD patient with CKD**?

For the purposes of this survey, please consider **systemic inflammation** to be an **hsCRP lab value of 2.0 – 10.0 mg/L.**

Please rank your top 3 reasons, with 1 being the top 3 reason, etc.

1. Proven clinical efficacy
2. Is affordable for patients
3. Is convenient for my practice
4. Is convenient for my patients
5. Is widely used for diagnosing inflammation
6. Has good insurance coverage
7. Current standard of care
8. Patients are willing to take this test
9. More sensitive than other available tests
10. Availability in the hospital or clinic lab
11. Recommended by local/institutional or international guidelines
12. hsCRP will influence my clinical decisions
13. Other, please specify:
14. Other, please specify:
15. Other, please specify:
16. I wouldn't use hsCRP for my patients with ASCVD <u>and</u> CKD

ALL QUALIFIED RESPONDENTS (S100r1)
Q410 Which of the following, if any, are reasons why you would **<u>not</u>** use **hsCRP testing** to diagnose **systemic inflammation** in an **ASCVD patient with CKD**?

For the purposes of this survey, please consider **systemic inflammation** to be an **hsCRP lab value of 2.0 – 10.0 mg/L.**

Please select all that apply.

1. Not familiar with this test
2. Patient reluctance/refusal
3. Not covered by health insurance
4. Out of pocket cost
5. Awaiting further diagnostic tests
6. Patient unable to tolerate blood draw
7. Not necessary given other tests available
8. Results are too sensitive
9. Typically do not test for systemic inflammation
~~10.~~ There are not any available treatments for systemic inflammation/will not change clinical outcomes
11. hsCRP variability
12. hsCRP will not influence my practice
13. Other, please specify:
99. There are no reasons why I would not use hsCRP testing

SELECT MORE THAN ONE REASON NOT TO USE hsCRP (Q410:2+responses, not 99)
Q415 Of the reasons you indicated for why you would **not** use **hsCRP testing** to diagnose **systemic inflammation**, what is your **top reason** for not considering the use of **hsCRP testing** in patients with ASVCD <u>and</u> CKD?

For the purposes of this survey, please consider **systemic inflammation** to be an **hsCRP lab value of 2.0 – 10.0 mg/L.**

1. Not familiar with this test
2. Patient reluctance/refusal
3. Not covered by health insurance
4. Out of pocket cost
5. Awaiting further diagnostic tests
6. Patient unable to tolerate blood draw
7. Not necessary given other tests available
8. Results are too sensitive
9. Typically do not test for systemic inflammation (hsCRP $\geq$2 mg/ L)
10. There are not any available treatments for systemic inflammation/will not change clinical outcomes
11. hsCRP variability
12. hsCRP will not influence my practice

SECTION 500: ASCVD MANAGEMENT ATTITUDES

ALL QUALIFIED RESPONDENTS (S100r1)
Q515 To what extent do you agree or disagree with the following statements

Please use a scale where "1" indicates "Strongly disagree" and "7" indicates "Strongly agree."

[COLUMNS]

Strongly disagree		Neither agree nor disagree				Strongly agree	Don't Know
1	2	3	4	5	6	7	9

1. I believe systemic inflammation is a risk factor to develop ASCVD.
2. I believe the level of systemic inflammation in the body impacts ASCVD prognosis.
3. I believe systemic inflammation is linked to the development of CKD.
4. I believe the level of systemic inflammation in the body impacts CKD prognosis.
5. I believe that the burden of systemic inflammation is higher in patients with both ASCVD and CKD.
6. I would like to learn more about the role of systemic inflammation in ASCVD.
7. Systemic inflammation measured by hsCRP is an indicator of ASCVD treatment efficacy.
8. Systemic inflammation is one the key drivers for cardiovascular events in patients with ASCVD and CKD.
9. Treating systemic inflammation in patients with ASCVD and CKD is crucial in my practice.
10. Residual inflammatory risk still persists even with availability of evidence-based preventive cardiovascular therapies for ASCVD with CKD patients at risk.
11. Ongoing chronic inflammation is an important contributor to the risk of recurrent cardiovascular event.
12. A lack of treatment options is the greatest unmet need facing patients with ASCVD and CKD.

ALL QUALIFIED RESPONDENTS (S100r1)
Q516 Next, we would like to understand what type of Cardiovascular guidelines (**LOCAL/INSTITUTIONAL** or **INTERNATIONAL)** are most influential to inform how you provide care for patients.

Please use a scale where "1" indicates "Local/Institutional guidelines are more influential", 4 indicates "Equally influential" and "7" indicates "International guidelines are more influential."

1 – Local/Institutional guidelines are more influential
2
3
4 – Equally influential
5
6

7 – International guidelines are more influential

ALL QUALIFIED RESPONDENTS (S100r1)
Q525 Now we would like to know more about how, if at all, you use Cardiovascular **LOCAL/INSTITUTIONAL** guidelines, position statements, and clinical practice updates to inform how you provide care for patients.

For which of the following topics of information do you typically reference **LOCAL/INSTITUTIONAL** guidelines, position statements, and clinical practice updates to inform your approach to care?

Please select all that apply.

1. Diagnosis of ASCVD
2. Management of ASCVD with pharmacological interventions
3. Management of ASCVD with non-pharmacological interventions
4. General information about CVD/heart disease
5. Heart failure
6. Dyslipidemia
7. Atrial fibrillation
8. Arrhythmia
9. Prevention/risk (for CVD in general)
10. Complications (with CVD in general)
11. Syncope
12. Antiplatelet Therapy (for CVD in general)
13. Hypertension
14. Hyperglycaemia
15. Obesity
16. Other, please specify:

96. I do not typically reference Cardiovascular LOCAL**/INSTITUTIONAL** guidelines for any reason

ALL QUALIFIED RESPONDENTS (S100r1)
Q526 Now we would like to know more about how, if at all, you use Cardiovascular **INTERNATIONAL** guidelines, position statements, and clinical practice updates to inform how you provide care for patients.

For which of the following topics of information do you typically reference **INTERNATIONAL** guidelines, position statements, and clinical practice updates to inform your approach to care?

Please select all that apply.

1. Diagnosis of ASCVD
2. Management of ASCVD with pharmacological interventions
3. Management of ASCVD with non-pharmacological interventions
4. General information about CVD/heart disease
5. Heart failure
6. Dyslipidemia

7. Atrial fibrillation
8. Arrhythmia
9. Prevention/risk (for CVD in general)
10. Complications (with CVD in general)
11. Syncope
12. Antiplatelet Therapy (for CVD in general)
13. Hypertension
14. Hyperglycaemia
15. Obesity
16. Other, please specify:
97. I do not typically reference Cardiovascular INTERNATIONAL guidelines for any reason

SECTION 600: OPPORTUNITIES AND EASTABLISHING TESTING AS SOC

ALL QUALIFIED RESPONDENTS (S100r1)
Q600 We would now like to understand how important various outcomes are in your consideration to assess systemic inflammation (hsCRP $\geq$2 mg/ L) in patients with ASCVD and CKD?

Please rate each outcome on the following scale:

Please use a scale where "0" indicates "Not at all important" and "10" indicates "Extremely important."

Not at all important										Extremely important
0	1	2	3	4	5	6	7	8	9	10

1. Proven clinical efficacy of testing
2. Testing is affordable for patients
3. Testing is convenient for my practice
4. Testing is convenient for my patients
5. Testing is widely used for diagnosing systemic inflammation
6. Testing has good coverage/low out of pocket costs for patients
7. Testing is well-tolerated by patients
8. Testing has good patient compliance
9. Testing is more sensitive than other available tests
10. Testing is available within my hospital or clinic
11. Testing is recommended by local/institutional or international guidelines
12. Availability of specific treatments to address systemic inflammation (hsCRP $\geq$2 mg/ L)
13. Wait times to receive results

ALL QUALIFIED RESPONDENTS (S100r1)

Q605 Below is the same list of outcomes that you just reviewed. Now we would like to understand how well these outcomes are currently being met with respect to assessing systemic inflammation (hsCRP $\geq$2 mg/ L) to enhance management of ASCVD.

Please rate each outcome to the on the following scale:

Please use a scale where "0" indicates "Not at all being met" and "10" indicates "Perfectly met."

Not at all being met										Perfectly met
0	1	2	3	4	5	6	7	8	9	10

1. Proven clinical efficacy of testing
2. Testing is affordable for patients
3. Testing is convenient for my practice
4. Testing is convenient for my patients
5. Testing is widely used for diagnosing systemic inflammation
6. Testing has good coverage/low out of pocket costs for patients
7. Testing is well-tolerated by patients
8. Testing has good patient compliance
9. Testing is more sensitive than other available tests
10. Testing is available within my hospital or clinic
11. Testing is recommended by local/institutional or international guidelines
12. Availability of specific treatments to address systemic inflammation (hsCRP $\geq$2 mg/ L)
13. Wait times to receive results

ALL QUALIFIED RESPONDENTS (S100r1)
Q610 Please rank the **top 3** most influential factors to establish assessment of high-sensitivity CRP (hsCRP) as **<u>standard of care</u>** in patients with ASCVD <u>and</u> CKD in your clinical practice?

Please rank your top 3 reasons, with 1 being the top 3 reason, etc.

1. Proven clinical efficacy of testing
2. Testing is affordable for patients
3. Testing is convenient for my practice
4. Testing is convenient for my patients
5. Testing is widely used for diagnosing systemic inflammation
6. Testing has good coverage/low out of pocket costs for patients
7. Testing is well-tolerated by patients
8. Testing has good patient compliance
9. Testing is more sensitive than other available tests
10. Testing is available within my hospital or clinic
11. Testing is recommended by local/institutional or international guidelines
12. Availability of specific treatments to address systemic inflammation (hsCRP $\geq$2 mg/ L)
13. Wait times to receive results

ALL QUALIFIED RESPONDENTS (S100r1)
Q615 What are the most common **unmet needs** faced by your patients with ASVCD and CKD?

Please rank your top 3 reasons, with 1 being the top 3 reason, etc.

1. Higher disease burden due to systemic inflammation
2. Higher risk of cardiovascular events and related complications
3. Limited biomarkers or tools to assess systemic inflammation
4. Limited awareness of role of systemic inflammation in ASCVD
5. Delay in screening for systemic inflammation
6. Delay in referrals to specialised clinical care
7. Delay early diagnosis and clinical management
8. Lack of effective treatment options for systemic inflammation
9. Polypharmacy: patients receiving multiple medications for disease management
10. Screening in primary care

96. Other, please specify: ____
97. Other, please specify: ____
98. Other, please specify: ____

SECTION 100: DEMOGRAPHICS

ALL QUALIFIED RESPONDENTS (S100r1)
Q105 Our final set of questions are for classification purposes only.

How do you describe yourself?

1. Male
2. Female
3. Gender Variant / Non-Conforming

98. Prefer not to answer

ALL QUALIFIED RESPONDENTS (S100r1)
Q110 In what year were you born?

Please enter as a four-digit number, e.g., 1963.

[_]_]_]_]

98. Prefer not to answer

ALL QUALIFIED RESPONDENTS (S100r1)
Q130 Which description best categorizes the practice setting where you work most often?

1. Urban
2.
